# LC-HRMS and Molecular Networking for Non-Targeted Screening of Emerging Contaminants in Cosmetics: Workflow, Applications, and Regulatory Challenges

**DOI:** 10.3390/molecules31152640

**Published:** 2026-07-29

**Authors:** Linrui Fu, Yike Song, Jie Zhu, Haiyan Wang, Yong Lu

**Affiliations:** 1 National Institute for Food and Drug Control, Beijing 100050, China; fulinrui357@163.com (L.F.); songyikele@163.com (Y.S.); 15684195373@163.com (J.Z.); summerwhy163@163.com (H.W.); 2 School of Light Industry, Beijing Technology and Business University, Beijing 100048, China

**Keywords:** emerging contaminants, cosmetic safety, molecular network, non-targeted screening, high-resolution mass spectrometry

## Abstract

Emerging contaminants (ECs) in cosmetics are introduced through various pathways, including illegal adulteration, raw material impurities, and packaging migration. Characterized by chronic exposure, potential toxicity, and an elusive nature, these contaminants pose a continuous and serious threat to public health. Currently, the regulatory control of ECs in the cosmetics industry is still evolving. Traditional targeted analysis, which relies heavily on predefined reference lists and standards, suffers from narrow coverage and fails to meet modern safety requirements for the comprehensive screening and early warning of ECs. To address this challenge, liquid chromatography–high-resolution mass spectrometry (LC-HRMS), combined with non-targeted screening (NTS) strategies and molecular networking (MN), serves as a powerful analytical tool. By operating in a full-scan mode without predefined targets, this technology comprehensively captures thousands of chemical features in cosmetic samples, enabling the prioritization and tentative annotation of concealed risk substances for subsequent structural confirmation. Furthermore, the spectral similarity clustering capability of MN facilitates the identification of homologous derivatives and structural analogs from a single known compound, significantly enhancing the efficiency and systematic discovery of ECs. From a regulatory perspective, this integrated approach provides crucial technical support for authorities to accurately assess risks and ensure consumer safety. Ultimately, it promotes a paradigm shift in cosmetic safety management, from a passive response to known hazards to the proactive prevention and control of emerging risks.

## 1. Cosmetics Safety and Challenges of ECs

ECs refer to a class of chemicals that can be detected in the environment and natural ecosystems, and even trace levels can pose risks to human health or environmental safety. ECs of wide concern at home and abroad mainly include persistent organic contaminants, endocrine disruptors, antibiotics, and microplastics [1], which generally have environmental persistence [2], biological activity [3], and potential ecological toxicity [4]. The impact of ECs on human toxicity and ecological environment has attracted the attention of the public and regulatory authorities. As one of the first signatories to the Stockholm Convention, China has carried out compliance activities in stages, regions, and industries to effectively control the environmental risks of ECs, such as persistent organic pollutants (POPs). The existing research on ECs is mainly based on ecological environment monitoring, and few studies have specifically assessed the risks related to ECs in cosmetics. However, ECs produced by cosmetics have been detected in the human body and the environment [5]. In view of the universality and necessity of cosmetics in people’s daily life, it is necessary and urgent to improve the effective screening of ECs in cosmetics.

The sources of ECs in cosmetics are hidden, complex, and multifaceted [6]. It can be disassembled from the core links between raw material ends, production ends, consumer use, and degradation processes, of which cosmetic raw materials are the most important source of ECs. Some plant extract raw materials may enrich ECs in the environment due to environmental pollution caused by plant growth and ultimately remain in cosmetics through the soil–plant cycle. It also includes contaminants affected by excipients and migration of cosmetics packaging materials. Cosmetic production processes may also be affected by improper process controls or derivatives mixed with ECs. After the use of cosmetics, chemical conversion may occur under the action of sweat, sebum, or ultraviolet light to form ECs. On 17 November 2025, the State Drug Administration issued the “Opinions on Deepening the Reform of Cosmetic Supervision and Promoting the High-quality Development of the Industry” (hereinafter referred to as “Opinions”) [7]. The “Opinions” put forward that, by 2035, the quality and safety supervision system of cosmetics will reach the international advanced level and basically realize the modernization of supervision. Strengthening the risk prevention and control of the whole chain of cosmetics is a strong driving force to promote the high-quality development of the cosmetics industry.

At present, the existing cosmetic detection methods are still missing for some new risk substances designed by chemical synthesis. High-resolution mass spectrometry (HRMS)-based NTS is a widely established strategy for the identification of ECs, including illegally added drug analogs, migrants from packaging materials, transformation products (TPs), and unknown by-products in raw materials. It can not only identify potential compounds by comparing databases but also detect unknown compounds [8,9]. High-throughput screening of ECs is the key to effectively evaluating the safety of cosmetic products. This technology has become an effective means to detect thousands of unknown chemical substances, but there are still limitations. For example, the identification and screening of unknown substances rely on existing databases, and there are major challenges in processing massive data generated by HRMS.

While a very recent and excellent review by Li, L. et al. [10] discussed the application of MN in cosmetics, their work predominantly focused on the computational frameworks, algorithmic foundations, and the exploration of natural active ingredients. However, a critical gap remains in the practical end-to-end analytical chemistry workflows specifically tailored for discovering ECs in highly complex cosmetic matrices.

**Figure 1:**
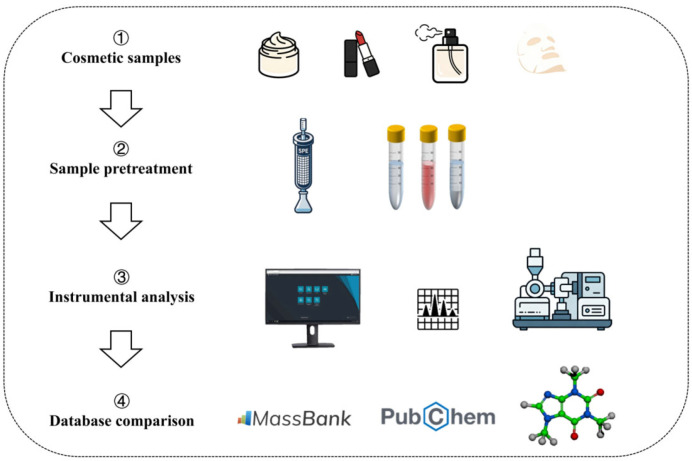
Cosmetic screening flow chart.

To explicitly differentiate our manuscript from the previous literature, the distinct novelty of this review lies in its comprehensive integration of the entire NTS analytical pipeline. Unlike previous works that mainly emphasize post-acquisition data processing, our review systematically addresses crucial pre-acquisition challenges, notably sample pretreatment strategies, and the construction of dedicated EC databases. Furthermore, we uniquely position MN not just as a computational tool but as a crucial downstream step within a holistic HRMS-based analytical methodology to tackle the specific safety challenges of ECs in cosmetics. A comprehensive analytical workflow for diverse cosmetic matrices, spanning from sample pretreatment to instrumental analysis and database annotation, is systematically summarized (Figure 1). Furthermore, to demonstrate the reliability and practical applicability of this approach, representative case studies of MN-based screening in real-world cosmetic testing are analyzed. Finally, we critically evaluate the current analytical limitations and propose future perspectives for the rapid screening and comprehensive monitoring of cosmetic safety.

## 2. Accelerating NTS via MN

### 2.1. Basic Principle of MN

MN technology [11] mainly relies on tandem mass spectrometry (MS/MS) data. The mass-to-charge ratio (*m*/*z*) and abundance of precursor ions (i.e., parent ions) were detected by primary mass spectrometry. Then, high-energy collisions occurred in the secondary mass spectrometry, the parent ions were fragmented into fragment ions, and the mass-to-charge ratio (*m*/*z*) of these ions was detected. Molecules with similar structures have similar stability and fracture modes of chemical bonds [12], so they will produce similar fragment ion spectra. According to the similarity of MS/MS spectra, MN transforms a large number of complex spectra into visual [13,14] network diagrams that intuitively describe the structural similarity between compounds.

As a frontier analysis strategy based on mass spectrometry fragmentation similarity, MN technology has been applied in many fields [10,15,16,17,18]. In the field of environmental science, MN technology has become a core tool for screening ECs and their TPs [19]. It can efficiently discover unknown TPs of drugs [20], antibiotics [21,22], and per- and polyfluoroalkyl substances (PFAS) [23] from complex matrices, such as wastewater, and significantly improve the efficiency and accuracy of non-targeted analysis, especially when screening in low abundance and with ECs.

Traditional non-targeted analysis methods can only identify one or two isolated ECs in the presence of homologs, TPs, and other ECs and may miss the powerful chemical family behind these. Based on the Global Natural Products Social (GNPS) MN [24] workflow and annotation tool [25], MN provides a new non-targeted screening strategy for the field of cosmetic safety through visualization capabilities, efficient clustering of structural analogs, and speculation of unknown structures. For example, when a known pollutant is highlighted in the network, the cluster connected to it can be regarded as a suspected area [26]. By analyzing the precise mass difference between nodes, it can be inferred whether an unknown is a homolog, isomer, or conversion product of the substance [27]. A known pollutant can lead to identifying a whole pollutant family, which greatly improves the efficiency of discovering ECs.

### 2.2. MN Types

In classic molecular networking (CLMN), its principle is to directly compare and cluster all MS/MS spectra in the original mass spectrometry data [28]. It is suitable for exploratory research, discovery of structural analogs of compounds, and preliminary visualization of ECs in cosmetic samples. The process is relatively simple and suitable for entry and preliminary exploration, but it cannot distinguish isomers and perform quantitative analysis, which has certain limitations in its application. Feature-based molecular networking (FBMN) [29] is a major evolution of CLMN. By introducing the key step of feature extraction [30], FBMN addresses several limitations, such as retention time and peak intensity, and facilitates relative feature abundance comparisons, which helps differentiate isomers that are chromatographically resolved. However, it is important to note that FBMN does not inherently resolve co-eluting isomers, stereoisomers, or isobaric species. Furthermore, while FBMN supports quantitative trends, absolute quantification still requires rigorous validation using calibration curves, internal standards, and matrix-effect corrections. The differences between FBMN and CLMN are shown in Table 1. Ion identity molecular networking (IIMN) is a supplement to the FBMN workflow proposed by Schmid et al. [31]. IIMN can identify and classify different ion forms derived from the same parent molecule to further refine the network.

### 2.3. MN in Cosmetics: Opportunities and Challenges

In summary, MN provides a breakthrough for the NTS of ECs and illegal additives in cosmetics. By visualizing chemical families based on MS/MS similarity, it effectively targets regulatory blind spots, excelling in the discovery of unexpected structural analogs, trace-level ECs, and degradation products in complex matrices without requiring reference standards. However, critical limitations remain for robust regulatory implementation. First, MN inherently struggles to distinguish structural isomers with identical fragmentation patterns. Second, existing open access databases (e.g., GNPS) lack sufficient coverage of cosmetic-specific ingredients and emerging environmental pollutants, which severely bottlenecks annotation accuracy and reporting confidence.

## 3. Analytical Methodology and Workflow

### 3.1. Construction of the EC Database

**Figure 2:**
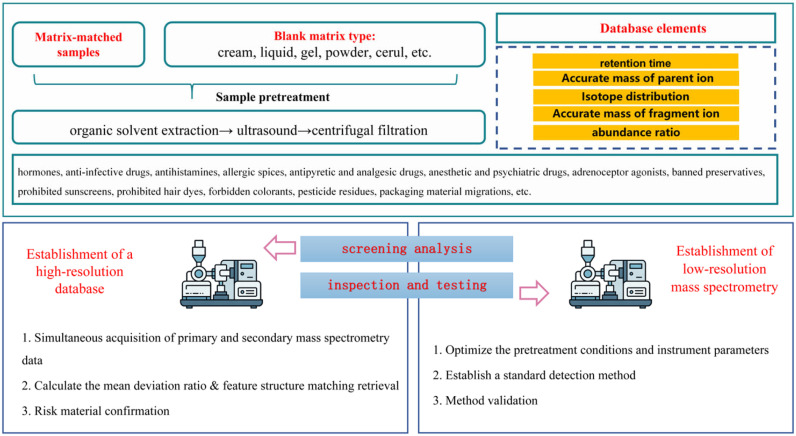
Establishment of a high-throughput screening database for ECs in cosmetics.

To bridge the gap between screening and confirmation, a standardized regulatory framework requires the establishment of comprehensive databases for priority ECs in cosmetics, typically by leveraging platforms like ultra-high-performance liquid chromatography–quadrupole time-of-flight tandem mass spectrometry (UPLC-Q-TOF-MS/MS). A critical strategy to ensure high data reliability and minimize the pervasive matrix effects in cosmetics is the adoption of a matrix-matched database establishment protocol (Figure 2). In this model, reference standards are systematically spiked into representative blank matrices and processed through uniform pretreatment procedures, such as ultrasound-assisted organic solvent extraction and centrifugal filtration, to ensure that spectral libraries reflect real-world analytical conditions. Representative efforts in this field have demonstrated that such rigorous workflows can yield robust digital libraries, which include retention times, accurate molecular weights, isotope distributions, and diagnostic fragment ions. By integrating these “electronic identification labels” into centralized safety platforms, the scope of monitoring can be expanded to cover diverse chemical classes—ranging from anti-infective drugs to packaging migrants and pesticide residues. Such centralized digital standard libraries serve as the technical backbone for proactive risk discovery, significantly enhancing the efficiency of regulatory agencies in identifying structural analogs and enforcing cosmetic safety standards.

Under this framework, the database should contain not only spectral signals but also a standardized set of compound-specific descriptors and metadata. The proposed database fields include compound names, CAS numbers and/or InChIKeys, regulatory statuses, exact masses, commonly observed adducts, retention times under defined chromatographic conditions, MS/MS spectra acquired at different collision energies, diagnostic fragment ions, isotopic patterns, source categories, confidence levels of annotation, and information on standard availability. These fields provide the necessary analytical and regulatory context for accurate compound matching, suspect screening, annotation confidence assessment, and subsequent confirmation. In particular, the inclusion of matrix-matched retention times, adduct behaviors, and fragmentation characteristics can improve the reliability of compound identification in complex cosmetic samples.

### 3.2. Sample Pretreatment

The matrix of cosmetics is complex, and the extraction results of sample pretreatment directly influences the following analysis and determination of results. Therefore, effective pretreatment must be carried out before analysis and detection [32]. Solid-phase extraction (SPE), as a supplement to liquid–liquid extraction (LLE), combines liquid chromatography and liquid–solid extraction technology to enrich, separate, and purify the target substances in cosmetic samples, which is more suitable for the analysis of liquid cosmetics [33]. As an efficient pretreatment technology for solid/semi-solid samples, the advantages of the matrix solid-phase dispersion (MSPD) method are highly compatible with the characteristics of non-liquid [34,35] cosmetics without additional liquefaction steps, significantly reducing the matrix effect. QuEChERS has significant advantages in the three fields of multi-component simultaneous analysis, complex matrix purification, and high-throughput detection due to its fast, simple, low cost, and high recovery characteristics. It is in line with the concept of modern green analytical chemistry and is suitable for the detection of cosmetics that are in direct contact with the human body [36,37]. Therefore, ensuring the matrix suitability of the sample pretreatment method is the core factor in cosmetic quality control, compliance verification, and scientific research innovation. In addition, for NTS applications, sample preparation should be evaluated not only in terms of analyte recovery and cleanup efficiency but also with respect to ion suppression, feature distortion, loss of unknown compounds, and source attribution uncertainty. A matrix-by-matrix summary of likely interferences, recommended extraction/cleanup strategies, and NTS-specific risks is provided in Table S1.

### 3.3. High-Resolution Mass Spectrometry Data Acquisition

In the field of non-targeted screening of cosmetics, data-dependent acquisition (DDA) and data-independent acquisition (DIA) are two mainstream mass spectrometry data acquisition strategies. In the precursor ions’ (MS1) full scan of DDA, several ions (parent ions) with the highest intensity were selected for fragmentation to obtain their secondary mass spectra. This is a selective process, in which high-abundance ions preferentially gain fragmentation opportunities [38]. In DIA, the entire mass range is divided into several windows without ion selection, and all ions in each window are fragmented and collected in an indiscriminate manner [39].

In the context of high-end research and increasingly stringent regulatory requirements, DDA and DIA are highly complementary rather than mutually exclusive (Table 2). Our laboratory uses the DIA model for large-scale sample screening to take into account the quality of the spectra and data integrity [40]. However, DIA inherently produces multiplexed (chimeric) spectra that require complex deconvolution, which can complicate MN. Therefore, DDA remains highly useful and indispensable for generating cleaner MS/MS spectra required for FBMN workflows and regulatory confirmation. For cosmetics, which are in direct contact with the human body and have high safety requirements, combining the panoramic view provided by DIA with the structural clarity of DDA can undoubtedly provide stronger security.

### 3.4. Integration Strategies for NTS in Cosmetic Analysis

**Figure 3:**
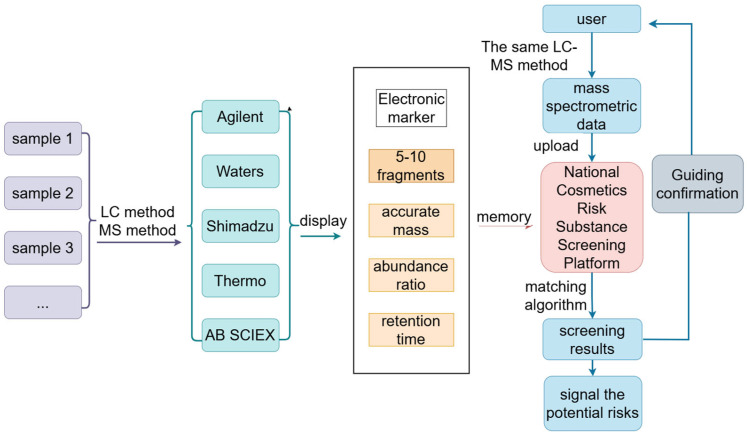
Application of cosmetic risk screening and early warning data platform.

The mass extraction window was set to 5 ppm for MS1 and 10 ppm for product ions (MS2). Chromatographic peaks were extracted from the full-scan total ion chromatogram (TIC), and peaks with recognizable analytical features were selected for subsequent evaluation. The acquired MS data from cosmetic samples were then matched against the database for screening and comparison, and the resulting candidate compounds were scored. When the matching score was greater than 0.6, the compound was reported as a suspect annotation. If the candidate further satisfied the structural interpretation criteria and screening rules described in Table 3 and Table 4 [41], it was assigned as a putative structure and prioritized as a potential risk substance in the sample. The screening result was then returned to the user for further evaluation (Figure 3).

It should be noted that database or public spectral library matches alone were not regarded as definitive identifications in this workflow. Instead, such results were reported as annotations or putative identifications, unless a higher confidence level was supported by additional evidence. Definitive confirmation of the target compound required subsequent verification using an authentic reference standard, including comparison of retention time and MS/MS spectral behavior under the same analytical conditions.

As illustrated in Figure 3, the proposed screening and early warning strategy operates through a closed-loop data platform. First, mass spectrometric data acquired from mainstream instruments (e.g., Agilent, Waters, and Thermo) were standardized into digital descriptors for risk compounds. Each descriptor functioned as an “electronic marker” defined by four key parameters: accurate mass, 5–10 diagnostic fragment ions, relative abundance ratios, and retention time. After user data were uploaded, the National Cosmetics Risk Substance Screening Platform automatically matched these markers and generated screening results. These outputs can support both rapid regulatory early warning and follow-up confirmatory analysis by laboratory users.

### 3.5. Data Processing and MN Construction

The data files (e.g., .raw, .d, .wiff) generated by mass spectrometers consisted of massive continuous signals, which recorded the mass-to-charge ratios and intensities of all ions detected over time. Due to the high dimensionality and complexity of these raw data, direct interpretation is extremely challenging. To accurately extract chemical information and construct a molecular network, a key step must be performed—data preprocessing [42].

#### 3.5.1. Data Preprocessing Steps

The goal of data preprocessing is to convert the original data into a clear molecular feature table. Each chemical characteristic comprises an accurate mass-to-charge ratio, retention time, and peak area. The pretreatment process is usually completed by professional mass spectrometry processing software, such as MZmine [43,44,45] MS-DIAL [46,47,48], XCMS [49,50], OpenMS [51], or Progenesis QI. Especially for cosmetic samples, this step is crucial to filter out high-abundance matrix interferences, such as PEG polymers and surfactants. The steps of data preprocessing [52] are as follows (Figure 3).

1. Preparation: This ensures that the original data (such as .raw, .d, .wiff format) have been converted to .mzML or .mzXML through the relevant software (such as MScovert).
2. Data importation (import files): The .mzML or .mzXML data format to be analyzed is imported into MZmine software.
3. Mass detection: This step identifies valid mass peaks within each scan by applying MS1 and MS2 noise thresholds to filter out background signals [53]. For centroided data (discrete data), algorithms rapidly extract *m*/*z*–intensity pairs based on peak height or area, making it highly efficient for high-throughput metabolomics [54]. For continuous profile data, curve-fitting models (e.g., Gaussian, Lorentzian) are employed to extract highly accurate (sub-ppm) mass information essential for precise qualitative analysis [55].
4. Chromatogram building: This step groups mass peaks with similar *m*/*z* across continuous scans to construct extracted ion chromatograms (EICs), forming the initial chemical features. Traditional algorithms like ADAP [56] achieve this by linking peaks in adjacent scans within a specified *m*/*z* tolerance. Furthermore, deep learning approaches like the DeepPIC model [57] now enable highly automated, cross-platform extracted ion chromatogram (EIC) construction for liquid chromatography–mass spectrometry (LC-MS) data.
5. Chromatogram deconvolution: As a core preprocessing step, deconvolution resolves overlapping peaks in the EIC and integrates them to extract exact retention times, peak widths, and areas (feature abundances). Algorithms like the local minimum search achieve this by identifying local maxima and defining peak boundaries based on intensity thresholds. Additionally, machine learning approaches, such as the MSHub tool developed by Aksenov et al. [58], have been introduced to automate deconvolution for gas chromatography–mass spectrometry (GC-MS) data.
6. Group isotopes and adducts: To prevent node redundancy in FBMN, isotopes and co-eluting adducts of the same compound must be merged into a single feature. Since GNPS relies on externally processed feature tables, this clustering is handled during preprocessing. Tools such as XCMS [59], MZmine, and MS-DIAL accomplish this by evaluating retention time consistency, peak shape correlations, and theoretical *m*/*z* shifts (or isotope ratios), ultimately outputting a streamlined, clean feature list for FBMN.
7. Peak alignment: This step matches identical compound features across all samples to generate a unified feature matrix. Highlighting its critical role in ensuring cross-sample comparability, Bai et al. [60] successfully utilized peak alignment to integrate multi-polarity data into a consistent matrix for FBMN construction.
8. Gap filling: This step addresses missing values in the LC-MS feature matrix caused by detection limits or alignment errors, ensuring stable downstream statistical analysis and network topology. Wei et al. [61] proposed a two-stage strategy combining K-Nearest Neighbors and random forests; this method employs local neighborhood information for initial estimation, followed by ensemble learning to refine predictions, thereby minimizing quantitative bias while preserving the original data distribution—a widely adopted practice in FBMN preprocessing.
9. Exportation for GNPS: Data is exported in .csv and .mgf formats for the FBMN workflow. The .csv file acts as the feature quantification table (containing peak areas, heights, RT, and *m*/*z*), while the .mgf file contains MS/MS spectra for computing spectral similarity. These paired files are critical for GNPS to accurately generate node attributes and construct the network [62].

**Figure 4:**
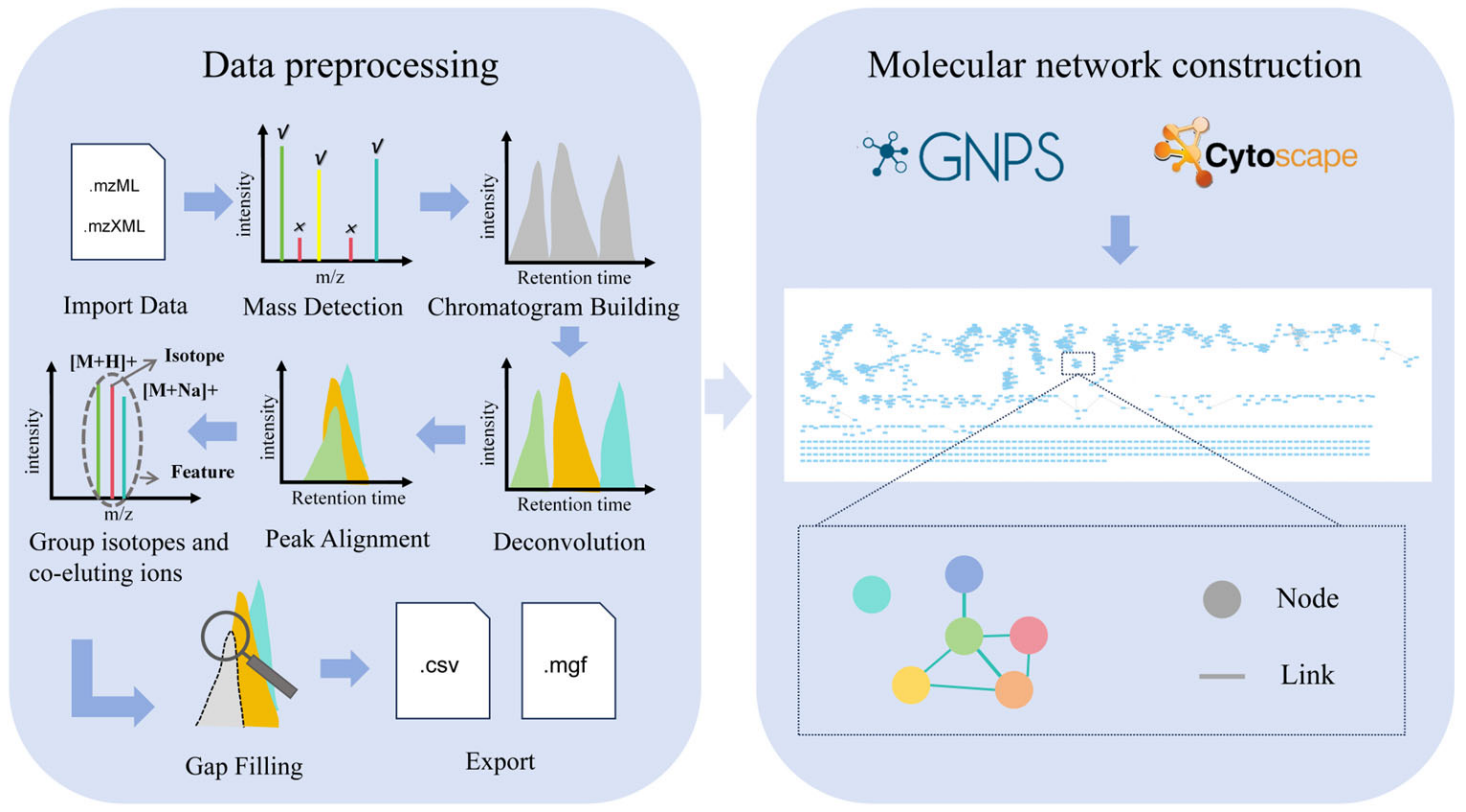
Molecular network construction process (In this schematic diagram, check marks (✓) and crosses (×) conceptually denote the retention of valid signals and the removal of background noise, respectively. Different colors are used solely as visual aids to differentiate distinct mass features or network nodes).

After completing the above series of data preprocessing steps (Figure 4), the following MN construction can be carried out.

#### 3.5.2. MN Construction: Taking FBMN as an Example

After data preprocessing, the next step is to use the GNPS platform to convert these data into a visual network. The construction process is as follows.

1. Data upload and workflow selection: The user uploads the preprocessed spectrogram .mgf and the feature table .csv files to the GNPS platform and selects the GNPS-FBMN workflow.
2. Spectral library search [63]: Concurrent with network construction, GNPS matches uploaded MS/MS spectra against public spectral libraries. Nodes exhibiting high similarity to reference spectra are automatically annotated with known compound identities. This process significantly accelerates the rapid identification of illegal additives, their derivatives, and ECs in cosmetic samples.
3. Molecular network construction: In this core step, GNPS computes pairwise similarities between all MS/MS spectra to construct the network, traditionally relying on the cosine similarity algorithm [64]. To optimize this process, Sheng et al. [65] evaluated four algorithms (modified cosine, entropy similarity, MS2DeepScore, and Spec2Vec), demonstrating that these methods exhibit distinct sensitivities to structural modifications. Their study highlights that strategically selecting or integrating multiple similarity algorithms can substantially enhance screening accuracy.
4. Key parameter settings: Following official GNPS recommendations and the FBMN protocol by Nothias et al. [64], key parameters are optimized to ensure data integrity. Typically, precursor and fragment ion mass tolerances are set to 0.01–0.02 Da and 0.02–0.05 Da, respectively, to maintain high matching accuracy. For network connectivity, a cosine similarity threshold (Min Pairs Cos) of ≥0.7 and a minimum of 6 matched peaks are required to filter weak connections, while the maximum number of neighbors (TopK) is limited to 10 to reduce network redundancy.
5. Network visualization and interpretation: The network can be visualized directly on GNPS or exported to Cytoscape [66] for advanced analysis. In the network graph, individual nodes represent specific features (with node size indicating abundance and labels reflecting library annotations), while edge thickness denotes structural similarity scores. Highly interconnected nodes form clusters that represent families of structurally related compounds, such as homologues, isomers, or metabolites [67].

#### 3.5.3. Strategy for Database Expansion: From Known Seeds to Unknown Targets

In summary, the collection of HRMS provides basic data for molecular networks. Through MN strategy, we can use the matched ‘seed’ compounds in the library to track their related unknown nodes (such as TPs). This process can transform unknown features into potential suspected targets, thereby conversely expanding our screening database and achieving a breakthrough from known to unknown.

### 3.6. Summary of the Analytical Workflow

In summary, the identification of ECs in cosmetics relies on a comprehensive workflow, where sample pretreatment, data acquisition, and data processing directly impact the node distribution in the subsequent molecular networks. Notably, adopting advanced pipelines such as FBMN significantly enhances annotation accuracy by incorporating MS1 features and retention times, effectively mitigating the inherent false-positive risks associated with traditional MN.

## 4. Case Study on the Application of MN in Cosmetics

### 4.1. Representative Case Studies in Cosmetic Analysis

To comprehensively evaluate the current state of NTS and MN, recent representative case studies were analyzed. As summarized in Table S2, these studies are critically compared across key performance dimensions, including matrix type, acquisition mode, networking workflow, and regulatory applicability. Notably, to address the diverse analytical needs, these workflows are explicitly categorized into “Direct cosmetic application” and “Methodologically transferable but not cosmetic matrix-validated” approaches.

In view of the detection of quinolones and illegal additives in cosmetics—that is, the traditional targeted method that has difficulty identifying unknown analogs and the pain points of lagging toxicity assessment—Yaqing Guo et al. [68] proposed a new screening strategy to obtain sample data through UPLC-Q-TOF MS/MS; integrate FBMN, quantitative structure–retention time relationship (QSIIR) modeling, and virtual toxicity prediction; and take quinolones as a case study. In the verification experiment, FBMN was used to cluster quinolones’ characteristic ion peaks from complex matrices to correlate known/unknown compounds. The QSIIR model used molecular structure parameters to predict chromatographic retention time and reduce qualitative false positives. Virtual toxicity prediction (such as ADMETlab) preferentially screened high-risk substances and reduced the amount of experiments. However, from a critical perspective, the predictive reliability of QSIIR models strictly depended on the structural diversity of the training set. When confronted with entirely novel synthetic scaffolds purposely modified to evade detection, the model’s accuracy may significantly decrease. Furthermore, purely in silico toxicity alerts require empirical validation before they can be confidently used for regulatory enforcement.

The study by M. Häßler et al. [69] aimed to evaluate the composition differences in different plant parts (leaves, flowers, fruits, stems) of Sambucus nigra by LC-HRMS and inductively coupled plasma optical emission spectrometry (ICP-OES), combined with multi-target and non-targeted screening, so as to provide a basis for screening and quality control of its medicinal parts. The strength of this study lies in its comprehensive profiling of both elemental and organic secondary metabolites, utilizing chemometrics to effectively identify specific botanical biomarkers. Nevertheless, the non-targeted organic characterization predominantly remained at a tentative identification level, highlighting the persistent challenge of confident structural elucidation in extremely complex botanical matrices. If such an approach were directly translated to finished cosmetic products containing these extracts, the interference from excipients (e.g., surfactants, emulsifiers) would inevitably cause much more severe ion suppression, making the detection of trace active ingredients or contaminants highly challenging.

In response to the health concerns caused by PFAS exposure in cosmetics, the Kelsey O’Malley [70] research team developed and validated a 5a-level suspected substance screening process (SSW) based on high-performance liquid chromatography–time-of-flight high-resolution mass spectrometry (HPLC-TOF-HRMS) for the detection of PFAS in 35 cosmetics in the US market. The process is based on a standard solution containing 30 typical PFAS, combined with a database containing 3882 PFAS from the National Institute of Standards and Technology (NIST) to achieve high confidence screening with low false positives and false negatives. This approach cleverly attempts to maximize screening coverage by matching mass features against a vast database (3882 PFAS), providing a much-needed high-throughput alternative given the severe shortage of commercial PFAS standards. However, relying predominantly on exact mass matching entails significant risks. Cosmetic matrices are rich in isomeric and isobaric compounds, which can easily lead to false-positive annotations when MS/MS fragmentation is not strictly required.

Suk Woo et al. [71] focused on the analysis of cosmetic colorants, particularly those belonging to chemical classes, such as azo and anthraquinone dyes, which are partially banned by multinational regulators due to health risks, such as carcinogenesis and mutagenesis. However, traditional liquid chromatography–tandem mass spectrometry (LC-MS/MS) detection is limited by the lack of standards, and it is difficult to cover non-targeted colorants. In order to solve the problem of simultaneous detection and confirmation of banned colorants in cosmetics, an analytical method combining MS/MS and molecular network technology was developed. In this study, the LC-MS/MS quantitative method was first established to realize the simultaneous analysis of 26 banned colorants. It was verified that the limit of detection (LOD) of the target in the samples of eyebrow tattoo, lipstick, and hair dye was 0.01–5.13 ng/mL, while the limit of quantitation (LOQ) was 0.03–15.39 ng/mL, which met the accurate quantitative requirements. A critical limitation, however, is the severe scarcity of high-quality MS/MS spectra for cosmetic colorants in public databases. Consequently, expanding the MS/MS spectral database to encompass a broader variety of dyes is essential for enhancing current annotation performance. At the same time, liquid chromatography–quadrupole time-of-flight mass spectrometry (LC-Q-TOF-MS) and MN technology are innovatively integrated to propose a non-targeted colorant-screening scheme. The scheme is based on spectral similarity. When the number of fragment ions ≥ 6 and the cosine score > 0.5, a characteristic molecular network map can be formed, and the structure and correlation of colorants can be inferred without standards. Through the analysis of the actual cosmetics samples spiked with three non-targeted banned colorants (*m*/*z* 267.116, 315.149, 345.157), banned components such as Disperse Red 17 and Disperse Red 1 were successfully identified, which verified the practicability of the method.

In order to solve the problem that TPs in the chlorination treatment of aromatic drugs and pharmaceuticals and personal care products (PPCPs) have not been deeply studied and analyzed due to the limitations of analytical techniques, the research team of Wen-Ling Chen et al. [72] developed a systematic screening and identification method based on HRMS to provide support for assessing aquatic environmental risks. The methodological strength lies in combining statistical filtering with isotopic pattern and fragmentation analyses to successfully trace hidden toxic derivatives that are more persistent and bioaccumulative than their parent compounds. Although this before-and-after comparative approach is highly effective in relatively clean aqueous systems, its direct application to cosmetic matrices would be problematic. The inherent batch-to-batch variations in complex cosmetic formulations would generate excessive false-positive variables in chemometric models. Additionally, the heavy reliance on manual expert interpretation of fragmentation pathways limits its scalability for routine high-throughput screening in cosmetics.

### 4.2. Critical Evaluation of Current Applications

In conclusion, the application cases discussed in this section underscore the transformative impact of MN in cosmetic safety and quality control. By leveraging LC-HRMS coupled with platforms like GNPS and FBMN, researchers can effectively tackle various complex matrices. The applications span multiple critical dimensions: (1) regulatory compliance, demonstrated by the rapid annotation of prohibited colorants and the discovery of unexpected, novel synthetic analogs of anti-infective drugs; (2) product efficacy, through the comprehensive characterization of natural active ingredients in botanical extracts; and (3) environmental safety, highlighted by the screening of persistent PFAS and the TPs of personal care chemicals. Ultimately, these diverse case studies validate that the multi-information MN strategy is not merely an analytical tool but a comprehensive screening framework essential for discovering hidden risks and ensuring the overall safety profile of modern cosmetics.

## 5. Ongoing Developments and Future Perspectives

### 5.1. Analytical Challenges Associated with Different Cosmetic Matrices and Sample Preparation

In order to achieve specific skin sensation, function, and stability, cosmetic formulations often contain multiple types of ingredients, and these complex ingredients can cause serious interference with pretreatment and detection analysis, which may not only conceal the characteristics of trace pollutants but also easily induce redundant connections in the network. Therefore, future research must focus on optimizing sample pretreatment strategies and strictly screening data pretreatment parameters to minimize matrix effects [73].

The goal of efficient purification technology is to maximize the removal of the matrix and retain the target, which requires highly customized strategies based on product types.

For simple water- or alcohol-based cosmetics, a simple “dilute-and-shoot” or generic SPE might suffice. However, for complex matrices, more aggressive treatments are needed. For example, oil-based products typically require the use of non-polar solvents like dichloromethane (DCM) as a primary extraction agent. DCM is essential to thoroughly dissolve the lipid-heavy matrix and ensure the complete release of hydrophobic contaminants that would otherwise remain trapped in the oil phase. In the case of pigmented or powder-based formulations, like foundations and blushers, the strong adsorption of organic analytes onto inorganic minerals necessitates high-energy ultrasonic-assisted extraction (UAE). This process is critical to disrupt the physical bonds between the contaminants and the solid particles, thereby ensuring recovery [74].

Furthermore, recent advancements have provided deeper insights into the mechanisms of interference removal and highly sensitive detection via LC-MS. Innovative sample preparation techniques and optimized ionization mechanisms have proven highly effective in mitigating matrix effects and enhancing the detection limits of target analytes [75,76,77]. These latest methodological improvements offer valuable references for optimizing extraction and detection efficiencies in complex cosmetic matrices.

### 5.2. Challenges in NTS Data Acquisition and Database Construction

For data acquisition, the complexity of cosmetic matrices necessitates a delicate balance between sensitivity and coverage. Choosing between DDA and DIA involves inherent trade-offs; DDA may miss low-abundance precursor ions due to intensity-based triggering, while DIA results in highly convoluted spectra that complicate deconvolution. Furthermore, the lack of standardized acquisition parameters—such as optimized collision energy (CE) and dynamic exclusion windows—often leads to inconsistent MS/MS fragmentation patterns across different HRMS platforms, hindering the repeatability of the experimental results [78].

Most existing high-quality MS/MS libraries (e.g., GNPS, MassBank, or NIST) are predominantly populated with metabolites, natural products, or environmental toxins. In contrast, cosmetic-specific substances remain severely underrepresented. Constructing a cosmetic-specific spectral database faces severe practical challenges. The massive volume of regulated substances and rapid updates in safety regulations impose immense pressure on the dynamic maintenance of these databases, causing static libraries to quickly become obsolete [79].

### 5.3. Future Prospects of MN

The deep integration of MN and NTS represents a crucial direction for promoting the accurate identification of compounds. MN is applied to complex spectral data to reveal the structural similarity between compounds in a higher dimension, enabling the clustering and rapid labeling of unknown substances. With the continuous improvement of the spectral libraries and the optimization of spectral algorithms, MN-based NTS will further improve the accuracy and comprehensiveness of compound identification and provide strong technical support for the rapid identification of ECs in cosmetics [80].

In addition, artificial intelligence (AI) and ML will play a central role in NTS. The spectrum prediction model based on deep learning will significantly expand the coverage of the mass spectrometry database and achieve efficient matching between experimental spectra and predicted spectra. The ML algorithm will help to automate the analysis process, from data preprocessing and feature extraction to results annotation, which can achieve intelligent and standardized processing. In addition, AI-driven toxicity prediction models will become an important tool for risk assessment, which can quickly infer potential biological toxicity based on chemical structures and provide early warning information for regulatory authorities [81].

With the improvement of computing power and the establishment of a large data platform, the whole process of NTS is expected to be automated, networked, and intelligent. By constructing an open and shared cosmetic ingredient database, combined with the self-learning and self-optimization capabilities of AI algorithms, the future NTS system will have stronger unknown identification capabilities and risk prediction accuracy, providing continuous technological innovation impetus for cosmetic safety supervision [82].

### 5.4. Current Limitations of the Approach

In cosmetic NTS, the absence of reference standards is an inherent limitation due to the unknown nature of the screening targets. Regulatory frameworks demand high certainty, reproducibility, and legal defensibility. Unlike targeted analyses, which rely on mature validation guidelines, NTS suffers from a lack of standardized verification protocols. Consequently, analyzing the same sample across different laboratories often yields highly inconsistent compound identifications, severely undermining the reliability of NTS for regulatory enforcement [83].

This lack of inter-laboratory standardization stems from critical variations at nearly every step of the NTS workflow. Differences in sample preparation strategies, varying instrumental platforms (e.g., Q-TOF versus Orbitrap), and disparate data acquisition modes (DDA versus DIA) [84] inevitably lead to highly divergent raw data profiles. To transition NTS from an exploratory screening tool to a legally defensible regulatory instrument for cosmetics, it is urgently necessary to develop harmonized analytical protocols, implement standardized quality control mixtures, and establish consensus-based guidelines for data processing and results reporting.

Beyond these standardization and regulatory hurdles, qualitative bottlenecks persist, particularly regarding isomer discrimination among prohibited substances [85]. While FBMN improves upon classical MN by integrating retention time with spectral similarity, accurately distinguishing isomers with highly identical chromatographic behaviors remains a formidable challenge. More critically, existing public databases are predominantly skewed toward natural products and metabolomics, leaving a critical void in exclusive spectral data for synthetic cosmetic raw materials, novel illegal additives, and their degradation products. To break through this qualitative bottleneck and improve screening accuracy, it is imperative to construct and share a comprehensive, cosmetic-specific mass spectrometry library.

## 6. Conclusions

In summary, the conventional safety supervision model for cosmetics is largely reactive, as it mainly depends on predefined lists of prohibited, restricted, or permitted substances and often responds only after consumer complaints or safety incidents emerge. However, the cosmetics industry is evolving rapidly, with continuous innovation in raw materials, formulation technologies, and packaging systems, which may introduce new and previously unrecognized contaminants. Under such circumstances, broader and more flexible analytical strategies are needed to complement traditional targeted monitoring.

LC-HRMS combined with MN provides a promising approach for non-target screening of cosmetics, as it enables comprehensive signal acquisition without relying entirely on predefined compound lists. In this framework, LC-HRMS offers accurate mass measurement and MS/MS fragmentation information, while MN facilitates the organization of structurally related features through spectral similarity, thereby supporting data interpretation and the discovery of related compounds. Together, these tools can improve the detection and prioritization of ECs in complex cosmetic matrices and can serve as an important complement to conventional targeted analysis.

Nevertheless, several challenges still limit the broader routine application of this strategy in cosmetic safety assessments. In particular, existing spectral resources are still insufficient, and many available libraries are general purpose rather than specifically designed for cosmetic matrices and contaminant profiles. Future progress in this field will require the establishment of harmonized acquisition methods, cosmetic-specific MS/MS libraries, and shared reference spectra to improve the comparability and reliability of screening results across different platforms and laboratories. In addition, matrix-specific validation, transparent confidence-level reporting, and stronger inter-laboratory reproducibility are essential for ensuring the robustness and regulatory usefulness of HRMS-based workflows.

Therefore, rather than being viewed as a replacement for conventional targeted methods, LC-HRMS combined with MN should currently be regarded as a powerful complementary strategy for broader contaminant surveillance and early risk discovery in cosmetics. Continued progress will depend on coordinated efforts in analytical methodology, computational tools, toxicological evaluation, and regulatory standardization.

## Figures and Tables

**Table 1:** Comparison of the difference between classical molecular networks and feature-based molecular networks.

Contrast Dimension	CLMN [28]	FBMN [29]
Core principle	The similarity of all collected MS/MS spectra was compared directly	Firstly, feature extraction is carried out, and then spectral similarity comparison is performed
Data processing flow	Simple	Relatively complex
Network diagram	Data redundancy, complex network diagram	Network diagram is clearer
Quantitative ability	No	Supports relative feature abundance; absolute quantification requires validation
Isomer discrimination	No	Improved when chromatographic separation is achieved; not sufficient for co-eluting or highly similar isomers

**Table 2:** Differences between DDA and DIA in non-targeted screening of cosmetics.

Comparison Metric	Data-Dependent Acquisition (DDA)	Data-Independent Acquisition (DIA)
Spectral purity	High—isolates specific precursor ions, yielding pure MS/MS spectra.	Mixed/low—co-fragments multiple precursors simultaneously.
Chimeric spectra	Rare (primarily occurs during near-isobaric co-elution).	Highly prevalent (inherent to the wide isolation window method).
Deconvolution burden	Low—precursor–fragment relationships are directly established by the instrument.	High—relies heavily on complex algorithms to assign fragments to precursors.
Library search compatibility	Excellent—clean spectra lead to high-confidence scoring against standard libraries.	Moderate—accuracy heavily depends on the success of software deconvolution.
Low-abundance coverage	Moderate—requires advanced methods (iterative DDA, exclusion lists) to avoid missing values.	Excellent—records all detectable ions indiscriminately, minimizing missed detections.
Reproducibility	Moderate—stochastic nature of top N selection can cause variations between runs.	High—continuous and systematic cycles ensure consistent data recording.
Network interpretability	High—gold standard for generating clear, accurate molecular networks without false edges.	Challenging—chimeric spectra can introduce false structural similarities in networks.
Suitability for regulatory confirmation	High—clear, unambiguous MS/MS evidence directly linked to specific precursors.	Moderate—requires rigorous data validation to prove precursor–fragment relationships.

**Table 3:** Conditions for target confirmation.

Discriminant Term	Judgment Conditions
Retention time	The absolute deviation of the retention time of the target and the reference standard under the same conditions was ≤0.2 min.
Mass accuracy	The relative deviation of *m*/*z* of the primary parent ion was less than 5 ppm. The *m*/*z* relative deviation of the secondary ion was less than 10 ppm.
Isotope peak	It was confirmed that the sample injection concentration can detect the main isotope peak and that *m*/*z* met the mass accuracy requirements of the target.
Relative ion abundance ratio of fragments	The main fragment ions were 2 and above, which met the mass accuracy requirements, and the relative ion abundance ratio met the maximum allowable deviation requirements for qualitative confirmation.

**Table 4:** Maximum allowable deviation of relative ion abundance in qualitative confirmation.

Relative Ion Abundance (k) k = (Intensity of Product Ion/Intensity of Base Peak) × 100%	k > 50%	50% ≥ k > 20%	20% ≥ k > 10%	k ≤ 10%
Maximum allowable deviation	±20%	±25%	±30%	±50%

## Data Availability

No new data were created or analyzed in this study. Data sharing is not applicable.

## Supplementary Materials

The following supporting information can be downloaded at: https://www.mdpi.com/article/10.3390/molecules31152640/s1, Table S1: Analytical challenges and corresponding sample preparation strategies for different cosmetic matrices in non-targeted screening (NTS); Table S2: The application of MN in non-targeted screening in cosmetics.

## Abbreviations

The following abbreviations are used in this manuscript:

ECs	Emerging contaminants
LC-HRMS	Liquid chromatography–high-resolution mass spectrometry
NTS	Non-targeted screening
MN	Molecular networking
POPs	Persistent organic pollutants
HRMS	High-resolution mass spectrometry
TPs	Transformation products
MS/MS	Tandem mass spectrometry
PFAS	Per- and polyfluoroalkyl substances
GNPS	Global Natural Products Social
CLMN	Classic molecular networking
FBMN	Feature-based molecular networking
IIMN	Ion identity molecular networking
UPLC-Q-TOF-MS/MS	Ultra-high-performance liquid chromatography–quadrupole time-of-flight tandem mass spectrometry
SPE	Solid-phase extraction
LLE	Liquid–liquid extraction
MSPD	Matrix solid-phase dispersion
DDA	Data-dependent acquisition
DIA	Data-independent acquisition
MS1	Precursor ions
MS2	Product ions
TIC	Total ion chromatogram
EIC	Extracted ion chromatogram
LC-MS	Liquid chromatography–mass spectrometry
GC-MS	Gas chromatography–mass spectrometry
QSIIR	Quantitative structure–retention time relationship
ICP-OES	Inductively coupled plasma optical emission spectrometry
SSW	Substance screening process
HPLC-TOF-HRMS	High-performance liquid chromatography–time-of-flight high-resolution mass spectrometry
NIST	National Institute of Standards and Technology
LC-MS/MS	Liquid chromatography–tandem mass spectrometry
LOD	Limit of detection
LOQ	Limit of quantitation
LC-Q-TOF-MS	Liquid chromatography–quadrupole time-of-flight mass spectrometry
PPCPs	Pharmaceuticals and personal care products
DCM	Dichloromethane
UAE	Ultrasonic-assisted extraction
CE	Collision energy
AI	Artificial intelligence
