# Supplementary material for: LC-HRMS and Molecular Networking for Non-Targeted Screening of Emerging Contaminants in Cosmetics: Workflow, Applications, and Regulatory Challenges
Source: Molecules. 2026 Jul 29;31(15):2640. doi: 10.3390/molecules31152640 (PMC13467950; doi:10.3390/molecules31152640)
Supplement: Supplementary file 1 [file molecules-31-02640-s001.zip › molecules-4288494-supplementary.pdf]

Table S1: Analytical challenges and corresponding sample preparation strategies for different cosmetic matrices in non-targeted screening (NTS).

Cosmetic matrix	Typical examples	Likely matrix interferences	Recommended extraction / cleanup	NTS-specific risks and considerations
Aqueous lotions	Toners, essences, sprays, makeup removers	Humectants (e.g., glycerol, propylene glycol), surfactants, preservatives, fragrances, ethanol/water-rich matrix components	Dilution and filtration for direct analysis when appropriate; SPE for enrichment and cleanup of trace analytes; LLE for moderately hydrophobic compounds	Highly abundant soluble additives may dominate MS signals; surfactants can cause ion suppression and unstable electrospray response; volatile compounds may be lost during concentration
Emulsions / creams	Moisturizing creams, lotions, foundations, cleansing creams	Lipids, waxes, emulsifiers, silicones, fatty alcohols, esters, thickening agents	Solvent extraction using acetonitrile, methanol, or mixed solvents; defatting by hexane partitioning or low-temperature precipitation; SPE cleanup; MSPD for semi-solid matrices	Severe matrix effects and ion suppression are common; co-extracted lipophilic background may generate redundant features; aggressive cleanup may remove unknown lipophilic contaminants together with matrix components
Sunscreens	Creams, sprays, gels, sticks containing UV filters	High concentrations of organic UV filters, inorganic particles (TiO_2, ZnO), oils, silicones, emulsifiers	Solvent extraction followed by centrifugation and/or filtration; cleanup to reduce dominant UV-filter background; additional particle removal for mineral sunscreens	Major UV filters can dominate precursor selection and MS/MS acquisition, masking low-abundance contaminants; inorganic particles may reduce extraction reproducibility; high analyte load may compress

Cosmetic matrix	Typical examples	Likely matrix interferences	Recommended extraction / cleanup	NTS-specific risks and considerations
				dynamic range
Lipsticks	Lipsticks, lip balms, lip glosses	Waxes, oils, pigments, dyes, flavoring agents, fragrances	MSPD or solvent-assisted extraction; mild heating when necessary to improve dispersion; dispersive SPE or defatting prior to LC-HRMS analysis	Wax-rich matrices may reduce extraction reproducibility; pigments and hydrophobic residues can contaminate the LC-MS system; highly nonpolar compounds may be underrepresented in LC-based NTS workflows
Powders	Loose powders, pressed powders, blushes, eyeshadows	Mineral fillers (talc, mica, silica), TiO_2, iron oxides, adsorptive particulate surfaces	Ultrasonic solvent extraction; MSPD; centrifugation and fine filtration to remove particulates; optional cleanup depending on pigment load	Adsorption of analytes onto particles may lower recovery; suspended solids can affect repeatability and instrument stability; inorganic-rich matrices may hinder recovery of certain analytes
Hair dyes	Oxidative and semi-permanent hair dye products	Aromatic amine precursors, couplers, oxidants, polymeric colorants, surfactants, strongly colored compounds	Solvent extraction under controlled pH; SPE cleanup; minimize exposure to heat, light, and air to reduce transformation; analyze fresh extracts whenever possible	Analytes may degrade or oxidize during preparation; artifact formation can complicate annotation; difficult distinction between original ingredients, degradation products, and reaction intermediates

Cosmetic matrix	Typical examples	Likely matrix interferences	Recommended extraction / cleanup	NTS-specific risks and considerations
Botanical extracts	Herbal cosmetics, plant-based serums, essential-oil-containing products, natural extracts	Polyphenols, terpenoids, alkaloids, chlorophyll, natural pigments, endogenous secondary metabolites	QuEChERS for broad extraction; SPE fractionation or polarity-based cleanup; dilution for highly concentrated extracts	Extremely high chemical diversity increases annotation difficulty; endogenous natural products may dominate molecular networks; higher risk of false-positive annotations and source misinterpretation
Packaging-contact products	Products stored in plastic tubes, pumps, laminated sachets, coated containers	Plasticizers, oligomers, antioxidants, photoinitiators, slip agents, adhesive-related migrants	Product extraction combined with packaging blank analysis; migration-oriented solvent extraction; strict use of procedural blanks and low-background labware	Difficult source attribution between formulation ingredients and packaging migrants; contamination from laboratory plastics may produce false positives; blank subtraction is essential for reliable interpretation

Table S2: The application of MN in non - targeted screening in cosmetics

NO.	Application Category	Analyte class	Matrix Type	Acquisition Mode	Networking Workflow / Strategy	Identification-Confidence Level	Standard Confirmation	Toxicity Prioritization	Regulatory Applicability	reference
1	Direct cosmetic application	quinolone analogues	Complex cosmetic matrices	UPLC-Q-TOF MS	FBMN - QSIIR - QSAR	High (Known/unknown structural correlation)	Partial (Validation for targets)	Yes (In silico virtual prediction, e.g.,	High (Pre-screens high-risk additives)	[68]

							ADMETlab)		
2	Direct cosmetic application (Botanical ingredients)	phenolic compounds	Botanical extracts	LC-HRMS	Chemometric analysis combined with MS profiling	Tentative (Non-targeted organic characterization)	Partial	No	Medium (Suitable for raw material QC, challenging for finished products)	[69]
3	Direct cosmetic application	PFAS	Finished US cosmetics	HPLC-TOF-HRMS	Suspect Screening Workflow (SSW) matching NIST database	Level 5a (Exact mass match)	Partial	No	Medium (High-throughput alternative, but risks false positives)	[70]
4	Direct cosmetic application	prohibited colorants	Eyebrow tattoo, lipstick tattoo, hair tint	LC-Q-TOF-MS	GNPS-based Molecular Networking	Tentative to Confirmed (Predicts non-targeted structures)	Yes (26 banned colorant standards)	No	High (Simultaneous quantitation & non-targeted screening)	[71]
5	Methodologically transferable (Not cosmetic-matrix validated)	Transformation Products (TP) of Aromatic PPCPs in	Aquatic environment	UPLC - Q - TOF	Statistical filtering + isotopic pattern + fragmentation	Tentative to Confirmed	No (Relies on expert interpretation)	No (Assesses ecological persistence, not direct	Low/Medium (Too much interference if applied	[72]

	Aromatic Pharmaceuticals and Personal Care)	analysis	cosmetic toxicity)	directly to cosmetic batch variations)
